# Fabrication of carbon nanomembranes by helium ion beam lithography

**DOI:** 10.3762/bjnano.5.20

**Published:** 2014-02-21

**Authors:** Xianghui Zhang, Henning Vieker, André Beyer, Armin Gölzhäuser

**Affiliations:** 1 Department of Physics, Physics of Supramolecular Systems and Surfaces, Bielefeld University, 33615 Bielefeld, Germany

**Keywords:** carbon nanomembranes, dissociative electron attachment, helium ion microscopy, ion beam-organic molecules interactions, self-assembled monolayers

## Abstract

The irradiation-induced cross-linking of aromatic self-assembled monolayers (SAMs) is a universal method for the fabrication of ultrathin carbon nanomembranes (CNMs). Here we demonstrate the cross-linking of aromatic SAMs due to exposure to helium ions. The distinction of cross-linked from non-cross-linked regions in the SAM was facilitated by transferring the irradiated SAM to a new substrate, which allowed for an ex situ observation of the cross-linking process by helium ion microscopy (HIM). In this way, three growth regimes of cross-linked areas were identified: formation of nuclei, one-dimensional (1D) and two-dimensional (2D) growth. The evaluation of the corresponding HIM images revealed the dose-dependent coverage, i.e., the relative monolayer area, whose density of cross-links surpassed a certain threshold value, as a function of the exposure dose. A complete cross-linking of aromatic SAMs by He^+^ ion irradiation requires an exposure dose of about 850 µC/cm^2^, which is roughly 60 times smaller than the corresponding electron irradiation dose. Most likely, this is due to the energy distribution of secondary electrons shifted to lower energies, which results in a more efficient dissociative electron attachment (DEA) process.

## Introduction

Carbon nanomembranes (CNMs) with monomolecular thickness and macroscopic lateral size represent a new type of functional two-dimensional (2D) materials [1]. A universal scheme to fabricate CNMs is the irradiation-induced cross-linking of aromatic self-assembled monolayers (SAMs), which allows for creating a variety of functional nanomembranes by using different molecular precursors as building blocks [2]. The properties of CNMs, such as stiffness, chemical functionality and porosity, can be tailored through a prudent choice of the molecular precursors and the fabrication conditions. CNMs are capable of being released from the substrate and transferred onto arbitrary substrates, e.g., solid supports and holey substrates [3]. Mechanical properties of freestanding CNMs were characterized by bulge test in an atomic force microscope (AFM): biphenyl-based CNMs possess a Young’s modulus of ca. 10 GPa and a remarkable tensile strength of ca. 600 MPa [4]. The possibility of transferring CNMs and their high mechanical strength make them suitable candidates for nanoelectromechanical systems (NEMS). Postsynthetic modifications, e.g., multilayer stacking [5], thermal annealing [6], chemical functionalization [7], and perforation [8–9], lead to a further tailoring of the performance of the CNMs and enable various investigations and applications.

The cross-linking of SAMs is so far conducted by exposure to electrons [10] and photons [11]. Electron irradiation induces the dissociation of C–H bonds at the phenyl rings. The consequent cross-linking between adjacent aromatic moieties is a critical step in the formation of CNMs. Both electron beam lithography and extreme ultraviolet (EUV) lithography have been utilized to fabricate CNMs from SAMs [11–12]. The EUV photon induced cross-linking is, for that matter, related to secondary electrons generated by the photoemission process [11]. Turchanin et al. investigated the electron induced cross-linking of biphenylthiol (BPT) SAMs on gold with complementary spectroscopic techniques and they suggested a dissociative electron attachment (DEA) as the dominating process to which both primary electrons and secondary electrons contribute [13]. However, a detailed picture of how the spatial distribution of cross-links evolves until a complete CNM has been formed is still missing.

Further modification and patterning of SAMs have been achieved by using ion irradiation (e.g. Ar^+^, Ga^+^, Si^+^, etc.), which leads to the desorption and the fragmentation of molecules [14–15]. High energy helium ions passing through polymer films modify the macroscopic properties of these films, too. This is related to changes in the chemical structures of the polymers [16–17]. Recently, the helium ion microscope (HIM) has been employed as an imaging and measurement tool for nanotechnology, for which the sub-nanometer sized ion probe and its resulting high brightness lead to a higher resolution and the small convergence angle of the ion beam leads to a larger depth of field. As an imaging tool, this instrument has a high surface sensitivity and is particularly advantageous to distinguish monolayers from the supporting substrate [18–19]. As a tool for nanofabrication, the low proximity effect that arises from the finite excited volume, in which the ion–material interaction takes place, extending deeply into the material, and the confinement of ion scattering to the secondary electron escape depth promise an outstanding performance of HIM [20]. So far, various approaches have been used to exploit the capabilities of HIM, such as ion milling [21], scanning helium ion beam lithography (SHIBL) [22], and helium ion beam induced deposition (HIBID) [20].

Here we used 4'-nitro-1,1'-biphenyl-4-thiol (NBPT) as a molecular precursor to form SAMs on a Au substrate and employed HIM both as a nanofabrication tool to cross-link SAMs and as an imaging tool for the ex situ observation of the crosslinking process. As regards the nanofabrication, both supported and freestanding CNMs were fabricated by transferring them onto a silicon substrate and a transmission electron microscopy (TEM) grid, respectively. As regards the investigation of the crosslinking process, the helium ion beam was programmed to irradiate NBPT SAMs with a series of different doses. The separation of cross-linked and non-cross-linked SAMs was conducted by transferring them onto a Si substrate with an oxide layer. The observation was done by using HIM in doing so taking advantage of the high surface sensitivity of the instrument.

## Results and Discussion

**Figure 1:**
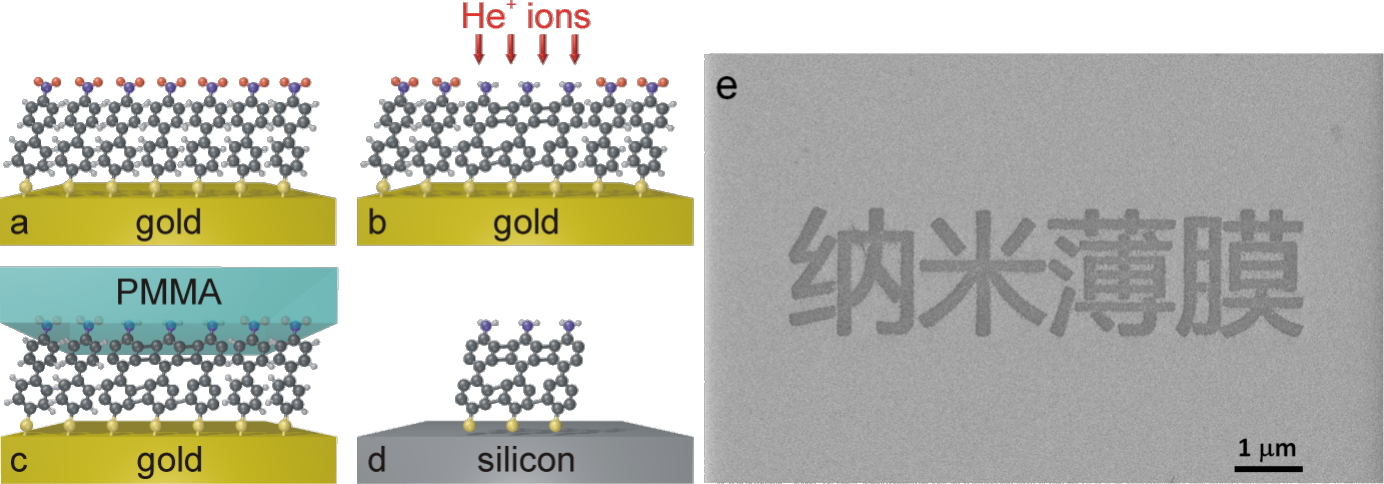
(a–d) A schematic representation of the NBPT SAM cross-linked with He^+^ ions and the transfer onto a Si substrate: (a) Formation of SAM on Au; (b) Local exposure to He^+^ ions; (c) Transfer of CNM with a polymeric film; (d) Separation of cross-linked from non-cross-linked regions. (e) A demonstration of lithographic CNMs in Chinese characters *nanomembranes* transferred on a silicon substrate.

Figure 1 shows a schematic representation of the cross-linking and transfer process. Firstly, the SAM that consists of closely packed NBPT molecules is formed on a gold substrate; secondly, the SAM is irradiated locally with He^+^ ions; thirdly, the transfer is assisted by a layer of poly(methyl methacrylate) (PMMA) for mechanical stabilization, which allows the dissolution of underlying Au layer; lastly, the PMMA layer is dissolved and only the cross-linked SAM is transferred onto another substrate, e.g., SiO_2_/Si. Figure 1e demonstrates a successful transfer of structured CNMs in Chinese characters which means *nanomembranes*: the grey background is SiO_2_/Si substrate and the darker features are transferred CNMs.

**Figure 2:**
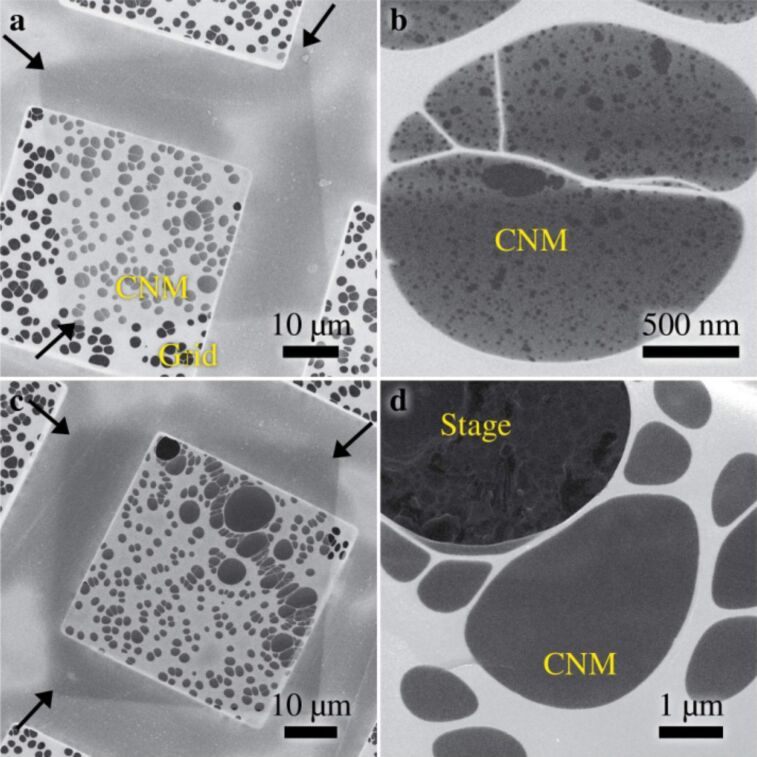
Freestanding CNMs with a dimension of 50 × 50 µm^2^ supported by a TEM grid with a holey carbon film: (a) the HIM micrograph of a CNM with an irradiation dose of 500 µC/cm^2^, where three arrows mark its corners; (b) the high magnification HIM micrograph shows that the CNM contains tiny holes; (c) the HIM image of a CNM with an irradiation dose of 1000 µC/cm^2^; (d) the high magnification HIM image shows that the CNM contains no microscopic defects. (Imaging doses: a) 0.27 µC/cm^2^, b) 55.9 µC/cm^2^, c) 3.36 µC/cm^2^ and d) 33.6 µC/cm^2^).

For the fabrication of freestanding CNMs, NBPT SAMs were irradiated in square patterns by helium ions at 35 keV and holey carbon-coated TEM grids were used as supporting substrates. Figure 2a shows the HIM image of a CNM with a size of 50 × 50 µm^2^ on a TEM grid and the corresponding irradiation dose is approximately 500 µC/cm^2^. The CNM is supported by a holey carbon film on a grid. The holey carbon film appears brighter and the CNM slightly darker due to the charging effect. To identify the CNM, its three corners are marked with arrows. Figure 2b shows the higher magnification HIM micrograph of the CNM in Figure 2a. It is noticeable that the CNM has many tiny holes, indicating that the crosslinking is not complete at this dose. Figure 2c and Figure 2d show the HIM micrographs of a CNM with an irradiation dose of ca. 1000 µC/cm^2^. The CNM shows homogeneous features and no pores are visible here, which indicates a complete crosslinking. The CNM in the upper–left corner is damaged and the dark features arise from the sample stage beneath the TEM grid (see Figure 2d). Notice that imaging doses are at least one order of magnitude smaller than the irradiation dose, no further modification of CNMs is expected during imaging.

**Figure 3:**
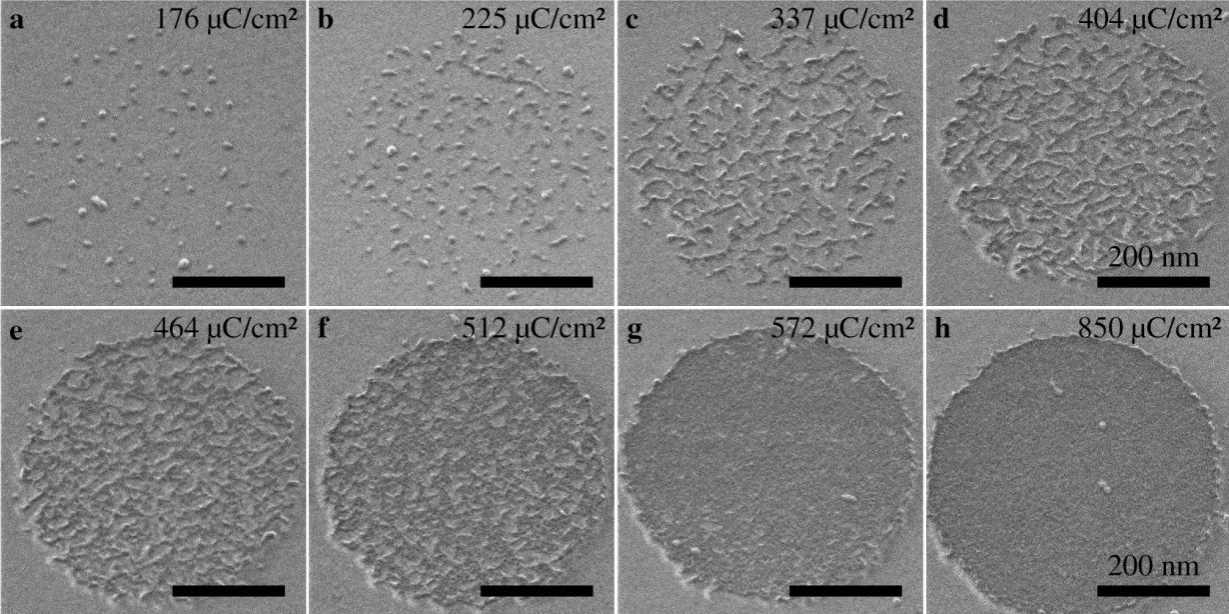
A series of HIM images showing the cross-linking of a NBPT SAM induced by helium ion irradiation, where the cross-linked SAM was transferred onto a SiO_2_/Si substrate after being cross-linked within a circular region with the dose given in the upper right corner of each image: (a) formation of circular shaped nuclei which are widely separated and randomly distributed. (b) more nuclei come into being and some of them start to grow one dimensionally; (c) chainlike structures with a typical length of ca. 100 nm become the majority; (d) chain thickening indicates a two-dimensional (2D) growth beginning to take place; (e–f) 2D growth plays a dominating role; (g) the CNM contains tiny holes; (h) the CNM forms completely and no defects are observed, indicating the status of a complete cross-linking. The scale bars are 200 nm.

In order to observe the development of the crosslinking of the SAM, the NBPT SAM was irradiated in circular regions by helium ion beam with a series of different doses. The variations of the irradiation dose are achieved by controlling the dwell time of the beam. Provided that the fabrication conditions are the same for the whole series, the irradiation dose is proportional to the total irradiation time. Therefore, the dynamics of cross-linking and growth regimes can be studied by making ex situ observations of the development of cross-linked SAMs. To this end, the distinction of cross-linked from non-cross-linked regions in the SAM was facilitated by transferring the irradiated SAM to a new substrate. Strictly speaking, there exists a threshold value, which is given by the density of cross-links that is required for a successful transfer of a monolayer. Below this threshold, the formed supramolecular network is not dense enough to sustain a lift-off from its initial substrate. Therefore, the transfer distinguishes the irradiated SAMs whose density of cross-links surpasses the threshold value from those below the threshold. Figure 3 shows a series of HIM images of a cross-linked SAM that have been transferred onto a SiO_2_/Si substrate. Interestingly, the first step is the formation of circular shaped nuclei, which is analogous to the nucleation for thin films or polymer crystallization [23]. After a dose of 176 µC/cm^2^ (Figure 3a), the average diameter of the nuclei is 9.0 ± 1.7 nm, which means that each nucleus consists of ca. 300 molecules, and the nucleus density is approximately 450 µm^−2^. When the dose is 225 µC/cm^2^, the nucleus density increases to approximately 930 µm^−2^ (see Figure 3b). The above mentioned threshold is related to the density of the cross-links of these smallest patches (nuclei) that are able to be transferred. After the early stage, the nuclei start to grow in one dimension and chainlike structures with a typical length of about 100 nm become the dominant features, as shown in Figure 3c. Figure 3d shows a marked change of structures, i.e., chain thickening, which indicates that a two dimensional (2D) growth (or lateral growth) begins to take place. Figure 3g and Figure 3h show an incomplete CNM with tiny holes and a complete CNM without holes, respectively. In order to make sure that all these structures are indeed CNMs and to exclude the possibility that some features (especially the small nuclei) might be due to contaminations from the transfer process, the sample (irradiated SAM on SiO_2_) was annealed up to 300 °C in ultra-high vacuum (UHV). The subsequent imaging with HIM confirms that no change of the structures occurs. It is also worth mentioning that an excessive exposure to He^+^ ions (>4000 µC/cm^2^) leads to a damage of the CNMs, which is attributed to the swelling of the Au substrate from ion implantation [24].

A complete cross-linking of NBPT SAMs by He^+^ ion irradiation requires an exposure dose of approximately 850 µC/cm^2^, which is roughly 60 times smaller than the corresponding electron irradiation dose (ca. 50,000 µC/cm^2^, 100 eV) [13]. The energy loss of helium ions in alkanethiol SAMs on Au were investigated by neutral impact collision ion scattering spectroscopy (NICISS) and the stopping power was determined to be about 3.7 eV/Å for the ion energy of 4 keV [25]. Though the total scattering cross–section of He^+^ ions by the SAM is very small, the energy transfer could induce molecular excitation and bond scissions, which may contribute to the cross-link formation to a certain extent. However, the tremendous dose difference can be associated with distinctive characteristics of secondary electrons that are excited by the helium ions. Firstly, the secondary electron yield for 35 keV He^+^ ions impinging perpendicularly on a Au substrate is calculated by the software package IONiSE to be about 2.7 [26]. And this is approximately three times higher than the experimentally determined secondary electron yield (approximately 0.85) for 100 eV electrons [27]. Secondly, the energy spectrum of secondary electrons excited by 35 keV He^+^ ions on Au showed a peak around 2 eV, with a small shoulder in the range of 5–6 eV [28]. For the excitation by electrons at 100 eV, the energy distribution of secondary electrons shows a peak at about 5 eV [27]. It is known that secondary electrons at energies well below the ionization threshold could produce single strand and double strand breaks in DNA and thus induce genotoxic effects in living cells [29]. These breaks are attributed to the DEA process, in which the attachment of incident electrons leads to the formation of a transient molecular anion (TMA) state and this TMA decays by electron autodetachment or by dissociation of a specific bond. The probability of forming a TMA, i.e., the electron capture cross section, varies inversely with the energy of the TMA state with respect to the ground state. In addition, the life time of TMAs increases with decreasing their energies [30]. This indicates that in the case of electron irradiation in NBPT SAMs, by analogy with strand-breaks in DNA, the DEA process is more efficient for secondary electrons with lower energies around 2 eV.

The DEA process is endothermic, as the electron affinity of a biphenyl molecule (3–7 kJ/mol) is much smaller than the bond energy of C–H (ca. 430 kJ/mol) [31–32]. The characteristic energy barriers for cross-linking arise from the activation energy for the DEA process and the entropic barrier to form a covalent bond among adjacent molecules. The activation energy and the above mentioned energy-dependent DEA cross section determine the rate coefficient of the DEA process [33]. The entropic barrier can be associated with a conformational entropy reduction of a molecule after being cross-linked, as a single molecule is more flexible and thus possesses higher degrees of freedom compared to a molecule being cross-linked and constrained by covalent bonds. The sequence of cross-linking depends on the characteristic energy barriers, as the entire region is irradiated homogeneously. A formation of nuclei would be associated with minimum activation energies in the SAMs. Further crosslinking prefers to occur around those already cross-linked nuclei, instead of regions that are not cross-linked. This implies that activation energies in cross-linked regions are relatively smaller, as π-electrons are laterally delocalized due to the cross-links and the electron mobility in cross-linked regions increases as well. The formation of interfaces between cross-linked and non-cross-linked region could result in entropic barriers due to steric hindrance. Note that the orientation of the 1D structures appears to be closer to the horizontal (scan) direction than to the vertical direction, which implies that the activation energy could be slightly brought down by the helium ion beam scanning due to the local electronic field around the growth front. Therefore, the growth direction of the nuclei is determined by the growth front that exhibits the lowest activation energies as well as the lowest entropic barriers. Lastly, the fact that a 2D growth follows the 1D growth could be attributed to a higher entropic barrier encountered at the sides of 1D structures. As regards the entropic barrier, an extreme example would be that for the insurmountable entropic barriers the crosslinking does not occur and vacancies that contain isolated molecules are formed. XPS spectra showed that the maximum degree of crosslinking of the BPT SAM was approximately 90% and further crosslinking was sterically hindered [13].

**Figure 4:**
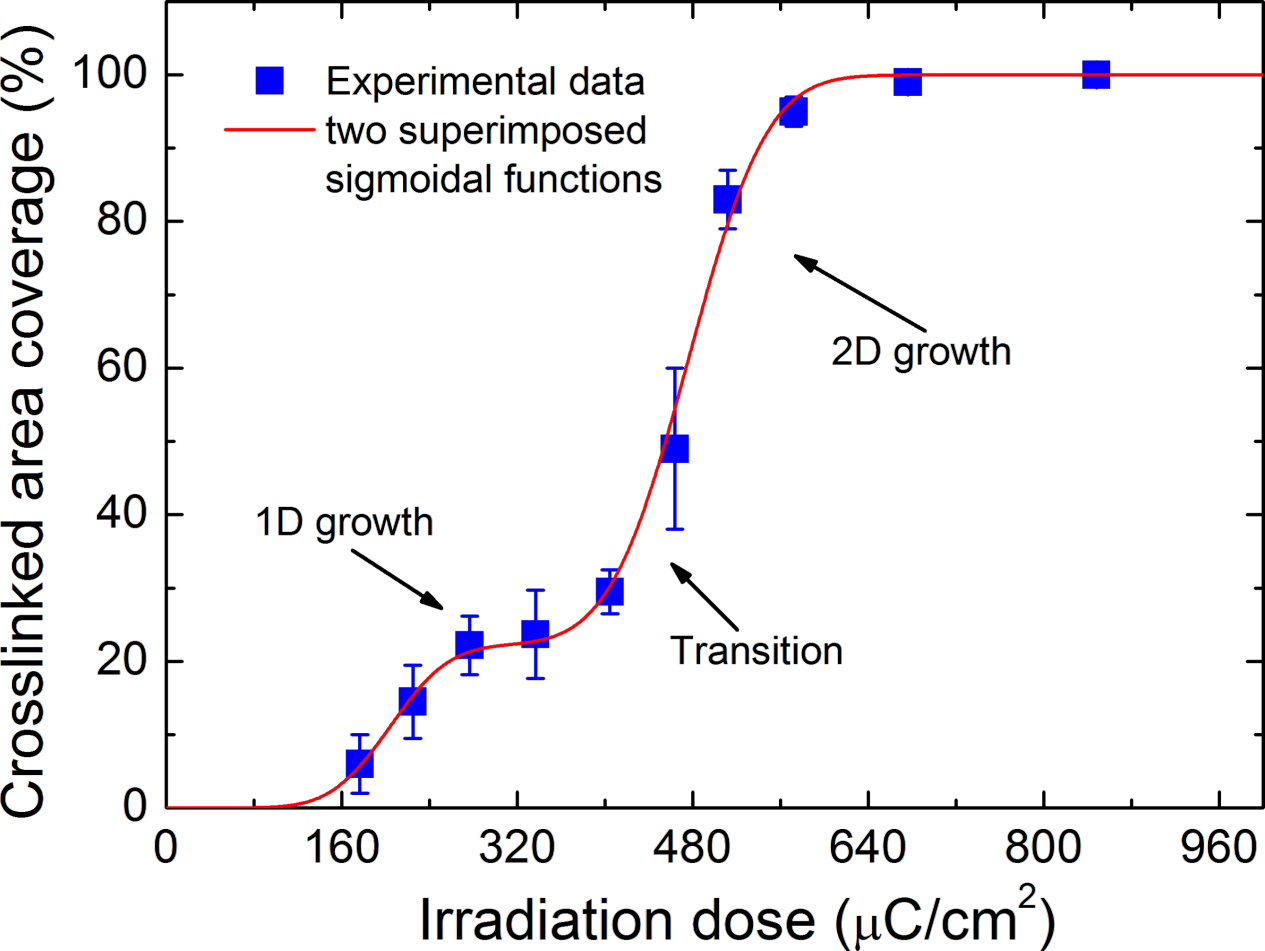
Percentage of the cross-linked area plotted as a function of the irradiation dose: (1) no CNM forms below the threshold dose of approximately 160 µC/cm^2^; (2) the formation of nuclei occurs up to a surface coverage of 6–10%; (3) the 1D growth dominates for a coverage of up to about 35% and the required mean dose is approximately 200 µC/cm^2^; (4) the 2D growth dominates for a coverage above about 35% and the required mean dose is approximately 480 µC/cm^2^.

As mentioned above, three stages of the crosslinking process were designated: the formation of nuclei, 1D growth, and 2D growth of cross-linked regions. Figure 4 shows the percentage of the cross-linked area as a function of the irradiation doses: (1) the initial formation of nuclei occurs up to a surface coverage of 6–10%; (2) the 1D growth dominates for a coverage up to about 35%; (3) the 2D growth dominates for a coverage above about 35%. We employed Gaussian distributions to describe the probability of surpassing the threshold cross-linking density at a given dose. As shown in Figure 4, the cross-linked area coverage in dependence on the exposure dose can be described by two superimposed sigmoid functions represented by the following cumulative Gaussian distribution functions

[1]
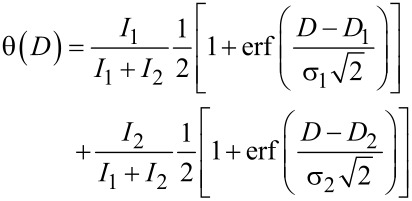


where θ is the cross-linked area coverage, *D* is the irradiation dose, *D*_1_ = (204 ± 18) µC/cm^2^ is the mean dose of the first Gaussian distribution, σ_1_ = (42 ± 24) µC/cm^2^ is the corresponding standard deviation, and *I*_1_ = 0.22 ± 0.04 is the magnitude of the first cumulative Gaussian distribution. The corresponding quantities of the second Gaussian distribution are *D*_2_ = (476 ± 8) µC/cm^2^, σ_2_ = (56 ± 7) µC/cm^2^ and *I*_2_ = 0.78 ± 0.04. The existence of two distinct Gaussian distributions indicates that two types of monolayer regions for 1D and 2D growth regimes are involved, which require different doses for cross-linking with mean values of approximately 200 µC/cm^2^ and approximately 480 µC/cm^2^, respectively.

A possible explanation for this behaviour can be found by considering the in-plane tension of cross-linked SAMs. It is known that free-standing CNMs from fully cross-linked NBPT SAMs exhibit an in-plane tensile residual strain of about 1% [4]. This strain is expected to be introduced during the cross-linking process, as new bonds are created between neighbouring molecules. Figure 3a shows the formation of small nuclei in the initial phase of the cross-linking process. Such island-like structures are known to partially relax compressive as well as tensile strain by slight expansion or shrinkage, respectively [34]. A 10 nm sized nucleus may shrink up to 1 Å by relaxing a tensile strain of 1%. Consequently, the distance between neighbouring molecules adjacent to such cross-linked nuclei should increase, which will reduce the probability of a new cross-link formation. This increases the mean dose for the non-cross-linked monolayer areas near cross-linked patches to reach the threshold cross-linking density. The two distinct Gaussian distributions in Figure 4 can be understood to reflect the cross-linking of unstrained and strained regions with mean doses of approximately 200 µC/cm^2^ and approximately 480 µC/cm^2^, respectively. A consequence of this interpretation is that the formation of nuclei as well as the formation of 1D structures is assigned to the cross-linking process with the lower mean dose, i.e., to the unstrained monolayer regions. This is obvious by Figure 3c and Figure 4. The HIM image of Figure 3c shows the occurrence of 1D structures while the second sigmoidal function in Figure 4 possesses a negligible value at this dose. Therefore, cross-linked patches are not isotropically surrounded by strained regions but in certain directions the adjacent monolayer is unstrained, which results in the observed formation of 1D cross-linked structures with the lower mean dose. The 2D growth of cross-linked areas is then assigned to the higher mean dose due to the strain in these monolayer regions.

## Conclusion

Freestanding carbon nanomembranes were successfully fabricated from aromatic self-assembled monolayers by using helium ion beam lithography. Three distinct stages of the crosslinking process, i.e., the initial nucleation, 1D growth and 2D growth, were observed ex situ by helium ion microscopy. Such a sequence could be related to different activation energies of dissociative electron attachment process as well as different entropic barriers encountered by the growth fronts. The irradiation dose for a complete cross-linking with helium ions is roughly 60 times smaller than that with electrons. Most likely, this is due to the energy distribution of helium ion excited secondary electrons being shifted to lower energies.

## Experimental

### Preparation of self-assembled monolayers

For the preparation of 4'-nitro-1,1'-biphenyl-4-thiol (NBPT) SAMs we used a 300 nm polycrystalline Au layer with (111) crystal planes epitaxially grown on a mica substrate (Georg Albert Physical Vapor Deposition, Germany). The substrate was cleaned with a UV/ozone cleaner (UVOH 150 LAB FHR) for 5 min, rinsed with ethanol, and then blown dry under a nitrogen stream. Afterwards the substrates were immersed into 10 mL of a solution of dry and degassed dimethylformamide (DMF) with ca. 10 mmol NBPT molecules for 72 h in a sealed flask under nitrogen atmosphere.

### Helium ion lithography and helium ion microscopy

The experiments were conducted with a Carl Zeiss Orion Plus^®^ helium ion microscope at room temperature. The irradiation of NBPT SAMs was performed by using the built-in software. The ion beam is programmed to irradiate an array of circular features by using a bitmap file and the dose variations are achieved by controlling the dwell time per pixel. The helium ion beam was operated at an acceleration voltage of 34.8 kV and a current of 3.5 pA. Due to the discreteness of bitmap files, the helium ion beam is intentionally slightly defocused in order to minimize any inhomogeneities in crosslinking. One circular feature consists of 2160 write points at a pixel distance of 10 nm. The fabrication of freestanding square CNMs was carried out by irradiating NBPT SAMs by HIM in a repeated scanning mode. The sizes of CNMs are the same to the field of view (FOV) and dose variations are achieved by controlling the total scanning time. For imaging, the helium ion beam was operated at acceleration voltages of 36.5–37.9 kV and currents of 0.3–0.6 pA. Images on SiO_2_ were acquired at a working distance of 9 mm and a tilt angle of 35° with 30 µs dwell time per pixel. Images on grid were acquired at a working distance of 30 mm with 0.5 µs dwell time and 128 frames averaged.

### Transfer of carbon nanomembranes

After helium ion irradiation, the whole NBPT CNMs were transferred onto another substrate for further investigations again with the HIM. For the transfer of NBPT CNMs onto a SiO_2_/Si substrate the samples were spin-coated with a layer of poly(methyl methacrylate) (PMMA) for stabilization and baked on a hotplate at 90 °C for 5 min. The separation of the PMMA/CNM/Au layer from the mica substrate was achieved by carefully dipping the sample into water. Subsequently, the Au layer was completely etched by a gold etchant (5 wt % I_2_ and 10 wt % KI in water). Afterwards, the PMMA/CNM layer was transferred to a Si substrate with an oxide layer with the thickness of 300 nm and the sample was immersed into acetone for 40 min for the dissolution of the PMMA layer. For the fabrication of freestanding NBPT CNMs on a TEM grid the same process was carried out, except for the drying process being conducted in a critical-point dryer (CPD, Autosamdri-815B, Tousimis, USA) to yield intact and suspended CNMs.
